# Bone cement implantation syndrome in cemented hip hemiarthroplasty—a persistent risk

**DOI:** 10.1007/s00068-020-01587-8

**Published:** 2021-01-26

**Authors:** Karoline Weingärtner, Philipp Störmann, David Schramm, Sebastian Wutzler, Kai Zacharowski, Ingo Marzi, Thomas Lustenberger

**Affiliations:** 1 Department of Trauma, Hand, and Reconstructive Surgery, University Hospital Frankfurt/Main, Theodor-Stern Kai 7, 60590 Frankfurt am Main, Germany; 2 Department of Anaesthesiology, Intensive Care Medicine and Pain Therapy, University Hospital Frankfurt/Main, Theodor-Stern Kai 7, 60590 Frankfurt am Main, Germany; 3 Department of Trauma, Hand and Orthopedic Surgery, Helios Dr. Horst Schmidt Kliniken Wiesbaden, Ludwig-Erhard-Street 100, 65199 Wiesbaden, Germany

**Keywords:** Bone cement implantation syndrome, Palacos reaction, Hip hemiarthroplasty, Risk factors, Outcome

## Abstract

**Background:**

Every year, ~ 210,000 initial implantations of hip endoprostheses are carried out in Germany alone. The “bone cement implantation syndrome” (BCIS) is considered a severe peri- and early-postoperative complication when implanting cemented prostheses. The origin of the BCIS and its impact on the clinical outcome are still uncertain. This study investigates the clinical progression after BCIS cases in patients with cemented hemiarthroplasty. Risk factors for the occurrence of BCIS are evaluated.

**Material and methods:**

Clinical data of all patients with a proximal femur fracture and which received a cemented hemiarthroplasty within a period of 9.5 years have been collected. BCIS (+) patients and BCIS (−) patients were compared with respect to their demographics and clinical outcome. Risk factors for the development of BCIS were identified.

**Results:**

A total of 208 patients could be included with complete data sets. The mean age was 81.1 ± 10.0 years. Overall, 37% of the patients showed symptoms of BCIS. In comparison to BCIS (−) patients there was a significantly higher rate of cardiovascular complications (27.3% vs. 13.7%, *p* = 0.016) and a higher in-hospital mortality rate (15.6% vs. 4.6%, *p* = 0.006) in BCIS (+) patients. Age, absence of a femoral borehole and ASA status were identified as statistically significant risk factors of BCIS.

**Conclusion:**

BCIS is frequently observed and in some cases severe complication. The therapy is exclusively symptomatic; identifying preventional measures might reduce the occurrence of BCIS.

## Introduction

In Germany alone, ~ 210,000 hip endoprosthesis are implanted annually. About 80% of these are due to coxarthrosis and ~ 13% for a proximal femur fracture [1]. Due to age and comorbidity, femoral neck fractures represent serious injuries in elderly patients and are associated with an increased risk of in-hospital complications [2, 3]. The bone cement implantation syndrome (BCIS), also called Palacos^®^ reaction, may be a serious intraoperative complication which is mainly observed when implanting a cemented prosthesis stem [4–6]. The incidence of BCIS varies widely in the literature between 28% up to 61.5%; this large variance is mainly due to the fact that there is no uniform definition of BCIS in the literature up to now [7, 8].

Clinically, the BCIS is associated with hypotension, increase or decrease in heart rate and decrease in oxygen saturation [5]. In severe cases, the increase of intramedullary pressure during cement placement may cause pulmonary embolism due to fat, bone marrow, air, or cement. The acute increase in pulmonary arterial resistance and the intrapulmonary shunt triggered by this can lead to right ventricular strain or even right heart failure and even death of the patient [9]. The BCIS has not yet been definitely explained. Among other things, a vasodilating effect of the cement is discussed as the cause of BCIS [10]. The release of polymerization gases, which occur during the exothermic hardening reaction of the cement, is also considered a possible cause. These gases are partially exhaled via the lungs, but can also lead to microembolism [11]. An anaphylactic, histamine-mediated reaction to the monomers was also demonstrated [12]. Prophylaxis with H1- and H2-blocking drugs, however, did not show any benefit [13]. The high intramedullary pressure measured during cementation and insertion of the prosthesis stem is probably a further cause for the formation and infiltration of embolisms. When using a cement gun, for example, peak intramedullary pressures of over 1100 mmHg can be detected [14]. A similar pathophysiological mechanism with an increased risk of bone marrow embolism can be observed during intramedullary nailing, during “reamer-irrigator-aspirator” (RIA) procedures, or even during the implantation of knee prosthesis.

The present study aims to investigate the influence of BCIS on the clinical course in patients with a proximal femur fracture and with implantation of a cemented hemiarthroplasty. Furthermore, risk factors for the occurrence of BCIS are to be determined.

## Material and methods

After approval by the Institutional Review Board (Nr. 497/16), we retrospectively reviewed all patients who presented with a femoral neck fracture and who received a primary endoprosthetic hip replacement with a cemented hemiarthroplasty in the Department of Trauma-, Hand- and Reconstructive Surgery at the University Hospital Frankfurt am Main from January 2010 to July 2019.

The data collected (from the time of admission to discharge) was extracted from the electronic patient file, the surgical reports and the anaesthesia protocols. The following patient characteristics were recorded: age, sex, diagnosis and previous illnesses. The severity of pre-existing conditions was determined using the American Society of Anaesthesiologists (ASA) risk classification [15]. Furthermore, hospital length of stay, postoperative complications and intra- and postoperative mortality were assessed.

With regard to surgery, the time of surgery, surgical technique (use of jet lavage, creation of a distal large-lumen femoral relief hole, use of an intramedullary plug) and the duration of surgery were recorded. The exact time of cement application was taken from the anesthesia protocols. The blood pressure values and oxygen saturation were determined before and after the application of the bone cement.

### Surgical technique

All procedures were performed under general anesthesia. As a standard, all patients received at least 500 ml of crystalloid infusion solution in the time between the induction of anaesthesia and the cementation of the prosthesis. Prior to the bone cement application, a pre-oxygenation with a FiO_2_ of 1.0 was performed and monitored by ECG, pulse oximetry and non-invasive blood pressure measurement. In patients with severe preexisting conditions, the monitoring was extended by invasive blood pressure measurement and measurement of the central venous pressure (CVP). Transesophageal or transthoracic echocardiography was only performed in emergency situations.

Only the product Palacos^®^ from Heraeus Medical was used as bone cement. The cement was mixed under vacuum with the suction of the polymerization gases. In all patients, a retrograde cement application with cement gun was performed.

Two different prosthesis stem types were used in the included patients, each combined with a bipolar femoral head: the MS-30 stem by Zimmer Biomet (Germany) and the IC long stem by Implantcast (Germany).

### Complications

Cardiovascular complications included all postoperative new cardiac arrhythmias (e.g. atrial fibrillation, atrial flutter, ventricular arrhythmias, AV blocks, etc.), myocardial ischemia (NSTEMI, STEMI), as well as hyper- and hypotension requiring therapy.

Only nosocomial pneumonia (hospital-acquired pneumonia, HAP) was included in the assessment of complications. Criteria for HAP included new, persistent or progressive infiltrates in combination with two of three other criteria: leukocytes > 10,000 or < 4000/μl, fever > 38.3 °C, purulent secretion [16]. The diagnosis was confirmed by appropriate imaging (X-ray, CT if necessary), the patient’s clinical symptoms, laboratory results and microbiology.

All pulmonary embolisms were diagnosed by CT morphology, or in the case of patients who died during surgery, by TEE/TTE, blood gas analysis and capnometry.

Acute renal failure was defined as follows: increase in serum creatinine by at least 0.3 mg/dl (26.5 μmol/l) within 48 h or increase in serum creatinine to at least 1.5 times a known or anticipated baseline within 7 days or decrease in urine excretion to < 0.5 ml/kg body weight/hour for at least 6 h [17].

### Study groups

The patients were divided into two groups: BCIS (+) (patients who showed symptoms of BCIS intraoperatively) vs. BCIS (−) (no symptoms of BCIS). The BCIS was defined based on the classification published by Donaldson et al. [18]:

Grade I: drop in oxygen saturation < 94%; drop in systolic blood pressure > 20%
Grade II: drop in oxygen saturation < 88%; drop in systolic blood pressure > 40%, or unconsciousness of the awake patient.
Grade III: cardiopulmonary resuscitation

### Statistical analysis

The data were entered in Microsoft Excel (version 2010); the statistical evaluation was carried out with “Statistical Package for Social Sciences” (SPSS for Mac, version 24.0, Chicago, IL, USA). The normal distribution was checked using the Kolmogoroff-Smirnov-Lilliefors test. The significance level was set at *p* < 0.05. All data are presented as mean ± standard deviation for continuous variables or as percentages for categorical variables.

The *T*-test or the Mann–Whitney *U* test was used to compare the continuously scaled variables. The categorically scaled variables were compared with the Chi-square test or the “Exact Fisher test”. A paired *T*-test was used to compare vital parameters before and after the cement application. To investigate the influence of BCIS on outcome parameters (complications, mortality), a logistic regression was performed. A stepwise logistic regression model was used to identify risk factors that were independently associated with the development of a BCIS and the risk factors from the bivariate analysis with a *p*-value of < 0.2 were integrated into the model.

## Results

**Fig. 1:**
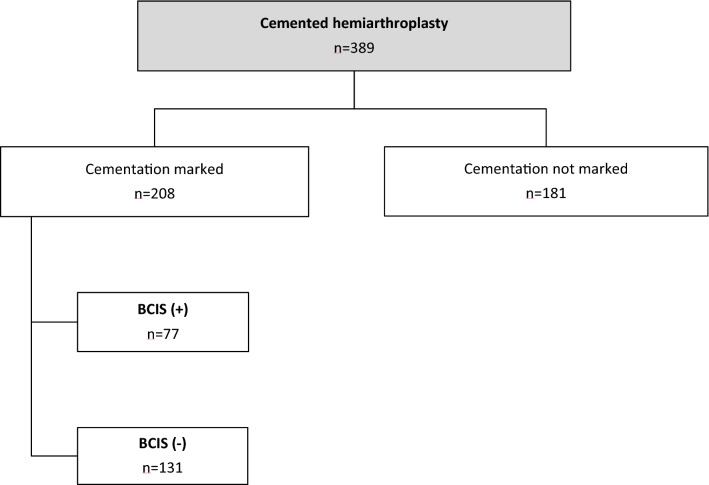
Flowchart

A total of 208 patients with complete data sets were included. Of these, 77 patients (37.0%) developed a BCIS. Overall, 53 patients (25.5%) had a BCIS Grade I, 17 patients (8.2%) a BCIS Grade II and 7 patients (3.4%) a BCIS Grade III (Fig. 1).

Table 1 shows demographic and surgery-related parameters of BCIS (+) and (−) patients. BCIS (+) patients were older (84.0 ± 8.2 vs. 79.5 ± 10.7 years, *p* = 0.003); no statistically significant differences were found regarding fracture localization and ASA status. In 10.1% of the cases, a long prosthesis stem was implanted, with no statistical differences between the two groups. A femoral borehole was applied in 28.4% (*n* = 59) and was performed significantly more frequently in BCIS (−) patients compared to BCIS (+) patients (34.4% vs. 18.2%, *p* = 0.012).

**Table 1:** Demographic parameters, injury characteristics and surgery-related parameters of BCIS (−) and BCIS (+) patients

	All patients *n* = 208	BCIS (−) *n* = 131	BCIS (+) *n* = 77	*p* value
Male	39.4% (82)	42.0% (55)	35.1% (27)	0.324
Age (years)	81.1 ± 10.0	79.5 ± 10.7	84.0 ± 8.2	0.003
subcapital femoral neck fracture	86.5% (180)	87.8% (115)	84.4% (65)	0.492
transcervical femoral neck fracture	2.9% (6)	2.3% (3)	3.9% (3)	0.672
pertrochanteric femur fracture	10.6% (22)	9.9% (13)	11.7% (9)	0.689
ASA 2 status	16.8% (35)	20.6% (27)	10.4% (8)	0.057
ASA 3 status	66.8% (139)	66.4% (87)	67.5% (52)	0.868
ASA 4 status	15.9% (33)	13.0% (17)	20.8% (16)	0.137
Operation specific parameters				
Duration of surgery (min)	97.3 ± 27.8	99.0 ± 29.2	94.5 ± 25.0	0.263
Jet-lavage	60.6% (126)	61.8% (81)	58.4% (45)	0.629
Femoral bore hole	28.4% (59)	34.4% (45)	18.2% (14)	0.012
Intramedullary plug	15.4% (32)	16.0% (21)	14.3% (11)	0.736
MS-30-stem	89.9% (187)	89.3% (117)	90.9% (70)	0.712
Long stem	10.1% (21)	10.7% (14)	9.1% (7)	0.712

### Hemoglobin (Hb) course and vital parameters

**Fig. 2:**
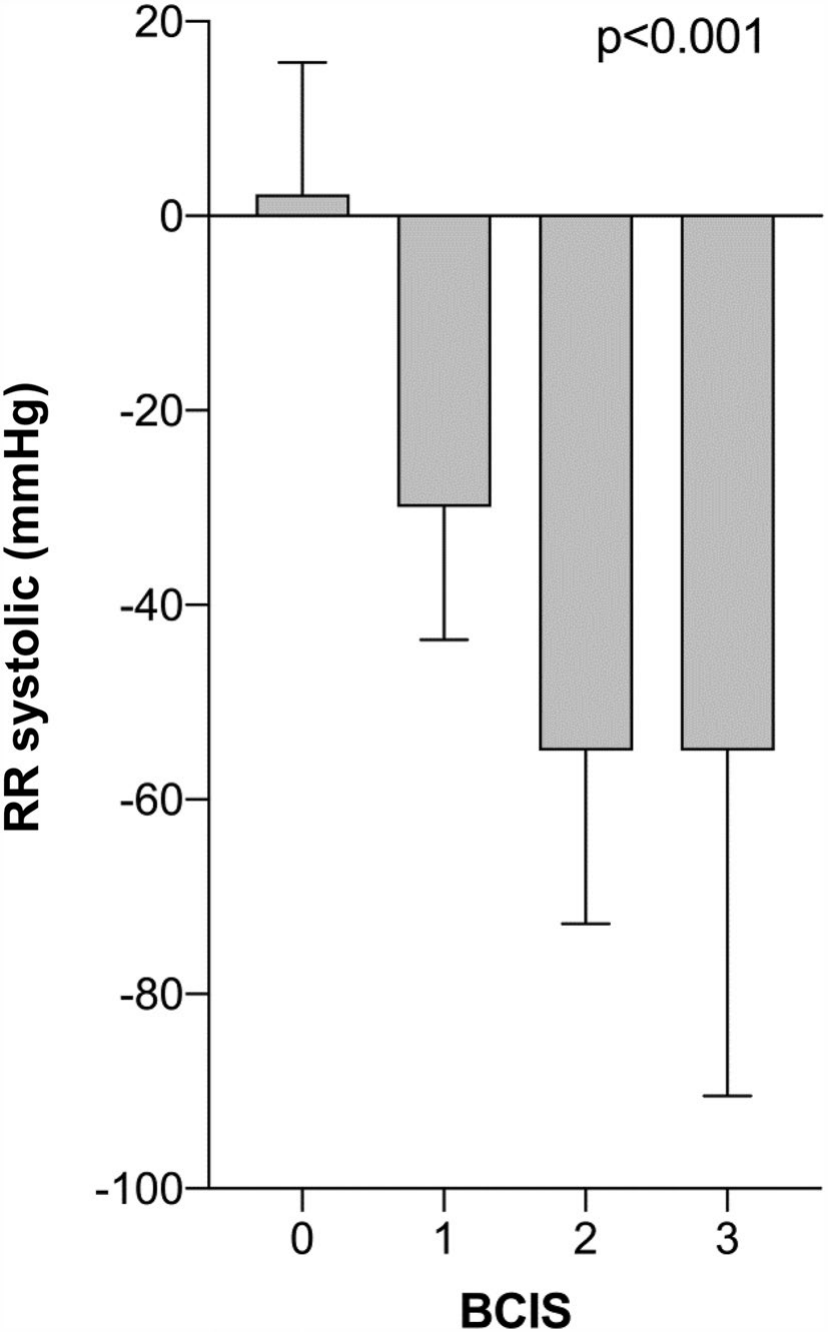
Change in systolic blood pressure during cement application stratified by BCIS severity. (Δ RR systolic = RR systolic after − RR systolic before cement application) (mean values ± standard deviation)

**Fig. 3:**
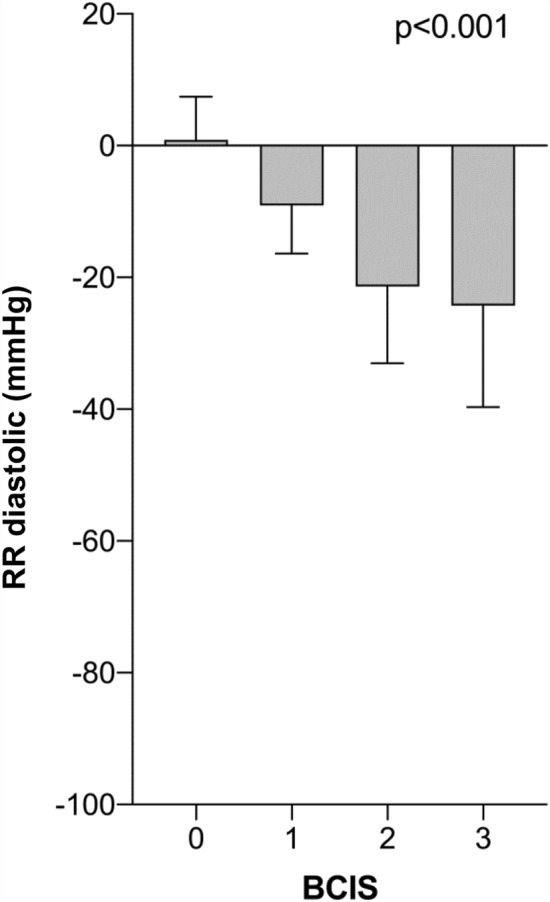
Change in diastolic blood pressure during cement application stratified by BCIS severity. (Δ RR diastolic = RR diastolic after − RR diastolic before cement application) (mean values ± standard deviation)

**Fig. 4:**
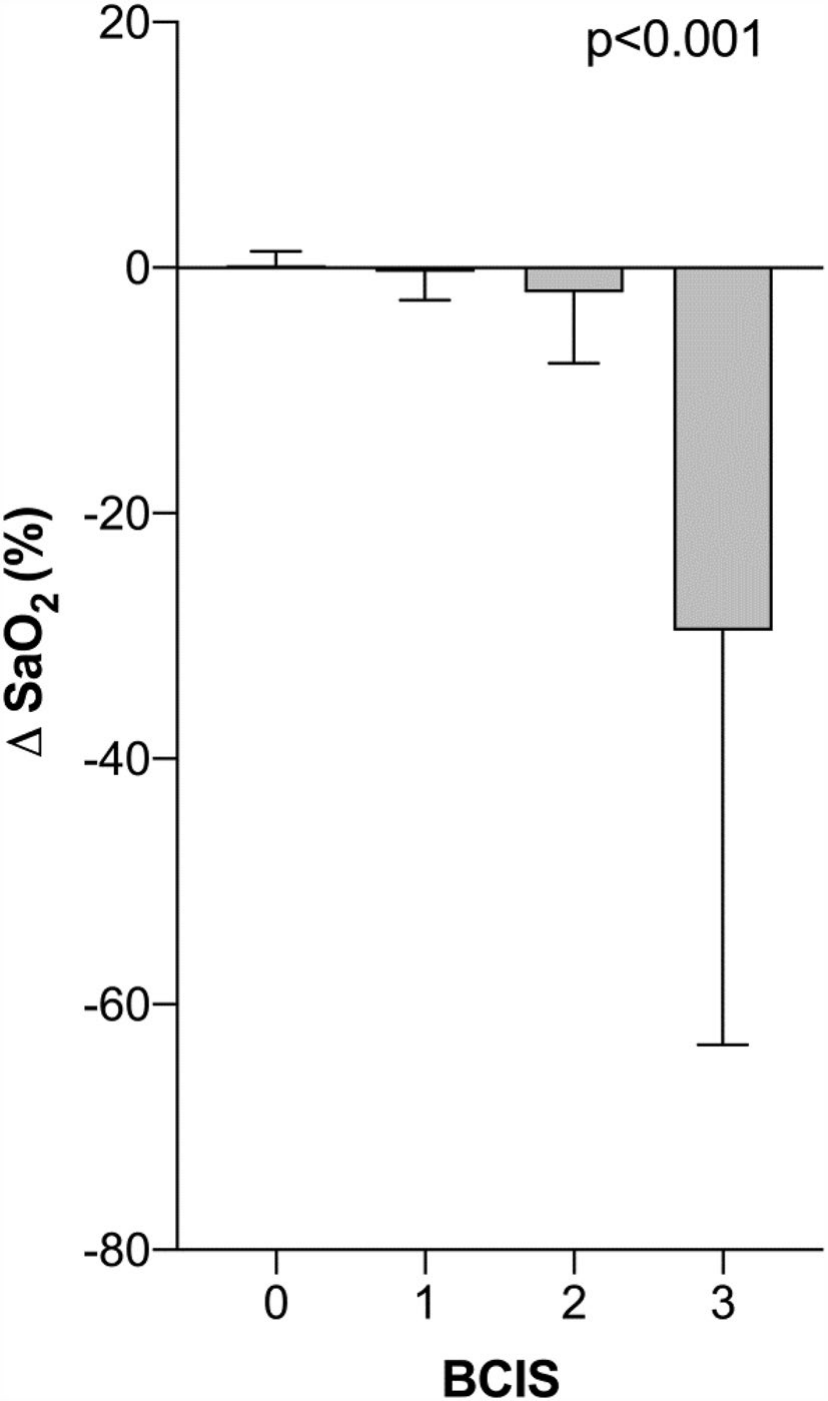
Change in oxygen saturation during cement application stratified by BCIS severity. (Δ SaO_2_ = SaO_2_ after – SaO_2_ before cement application) (mean values ± standard deviation)

Both pre- and postoperatively, there were no differences between the two groups with regard to Hb values (Table 2). Comparing preoperative and postoperative Hb values using a paired *T*-test, significantly lower Hb values were found postoperatively in the BCIS (−) and BCIS (+) groups. In the BCIS (+) group, significantly lower SaO_2_, systolic and diastolic blood pressure values were likewise found after cement application. With increasing BCIS severity, SaO_2_ values as well as systolic and diastolic blood pressure values (BCIS definition according to Donaldson et al.) significantly decreased (Figs. 2, 3, 4).

**Table 2:** Hemoglobin values pre- and postoperatively and vital parameters immediately before and after cement application

	All patients *n* = 208	BCIS (−) *n* = 131	BCIS (+) *n* = 77	*p* value
Hb preoperative (g/dl)	12.3 ± 1.8	12.3 ± 1.8	12.5 ± 1.7	0.521
Hb postoperative (g/dl)	8.1 ± 1.2	8.2 ± 1.3	8.1 ± 1.2	0.813
(Paired *T*-test) *p* value		< 0.001	< 0.001	
SaO_2_ before cement application	98.4 ± 1.6	98.4 ± 1.6	98.4 ± 1.5	0.83
SaO_2_ after cement application	97.3 ± 8.2	98.6 ± 1.5	95.0 ± 13.1	0.002
(Paired *T*-test) *p*-value		0.035	0.027	
RR systolic before cement application (mmHg)	118.3 ± 17.3	115.0 ± 13.4	124.0 ± 21.4	< 0.001
RR systolic after cement application (mmHg)	105.7 ± 22.2	117.2 ± 14.6	86.2 ± 19.0	< 0.001
(Paired *T*-test) *p*-value		0.065	< 0.001	
RR diastolic before cement application (mmHg)	58.8 ± 9.2	57.3 ± 8.5	61.4 ± 9.8	0.002
RR diastolic after cement application (mmHg)	54.5 ± 10.6	58.1 ± 8.4	48.3 ± 11.2	< 0.001
(Paired *T*-test) *p*-value		0.134	< 0.001

### Outcome

A total of 78 patients (37.5%) showed a postoperative irregularity in the clinical course (BCIS (−) 33.6% vs. BCIS (+) 44.2%, corr. *p* = 0.209) (Table 3). BCIS (+) patients showed a significantly higher rate of cardiovascular complications compared to BCIS (−) patients (27.3% vs. 13.7%, corr. *p* = 0.038).

**Table 3:** Intra- and postoperative complications in BCIS (−) and BCIS (+) patients

	All patients *n* = 208	BCIS (−) *n* = 131	BCIS (+) *n* = 77	*p*-value	Adj *p*-value*	Adj. OR (95% CI)*
Complications (all)	37.5% (78)	33.6% (44)	44.2% (34)	0.128	0.209	0.69 (0.38–1.24)
Cardiovascular complications	18.8% (39)	13.7% (18)	27.3% (21)	0.016	0.038	0.47 (0.23–0.96)
Pulmonary embolism	2.9% (6)	0% (0)	7.8% (6)	0.002		
Arterial hypotension	3.8% (8)	2.3% (3)	6.5% (5)	0.149		
Thrombosis	1.0% (2)	0.8% (1)	1.3% (1)	1.0		
Respiratory failure	6.3% (13)	6.9% (9)	5.2% (4)	0.771		
Pleural effusion	7.2% (15)	7.6% (10)	6.5% (5)	0.759		
Gastrointestinal complications/kidney failure	11.1% (23)	9.2% (12)	14.3% (11)	0.255	0.113	0.44 (0.19–1.19)
Acute abdomen	1.4% (3)	0.8% (1)	2.6% (2)	0.556		
GIT bleeding	1.0% (2)	0.8% (1)	1.3% (1)	1.0		
Kidney failure	9.1% (19)	8.4% (11)	10.4% (8)	0.630		
Infections	13.5% (28)	14.5% (19)	11.7% (9)	0.566	0.56	1.30 (0.55–3.09)
Pneumonia	10.1% (21)	9.9% (13)	10.4% (8)	0.914		
Wound infection	3.4% (7)	3.8% (5)	2.6% (2)	1.0		

*p value/OR multiple regression, corrected for age

**Fig. 5:**
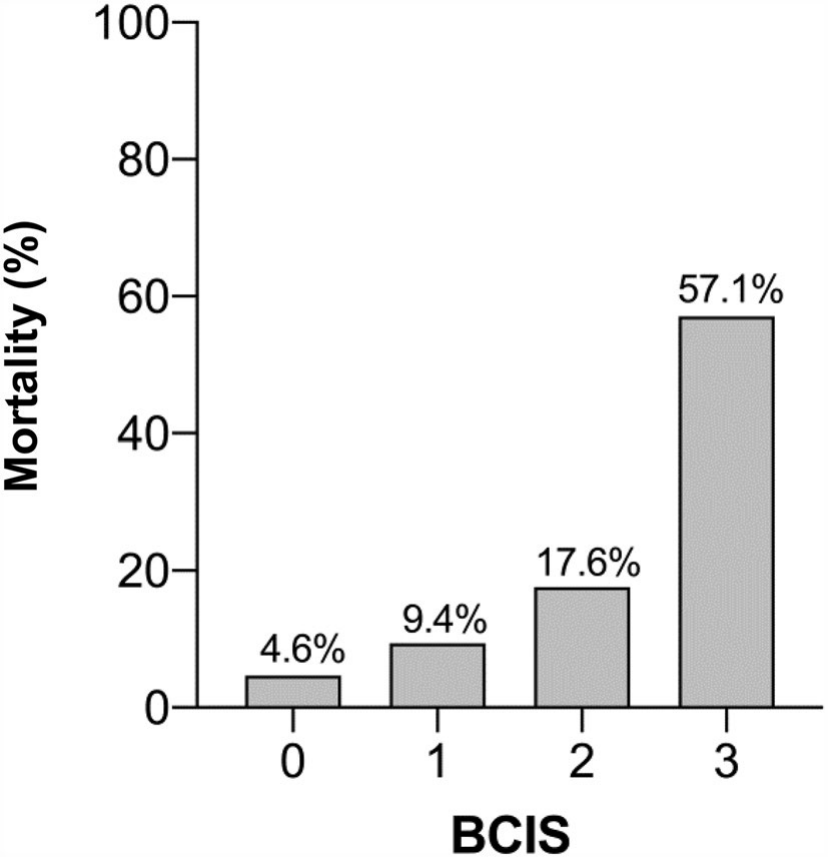
In-hospital mortality stratified by BCIS severity

Patients with BCIS (+) showed a statistically increased in-hospital mortality rate compared to BCIS (−) patients (15.6% vs. 4.6%, adj. *p* = 0.008) (Table 4). Postoperative mortality was 7.2% overall and was higher in the BCIS (+) group (11.7% vs. 4.6%, adj. *p* = 0.047). With increasing BCIS severity, a significant increase in mortality was observed (*p* < 0.001) (Fig. 5). No statistically significant differences were found with regards to the overall and postoperative length of hospitalization comparing the BCIS groups.

**Table 4:** In-hospital mortality and hospital length of stay in BCIS (+) and BCIS (−) patients

	All patients *n* = 208	BCIS (−) *n* = 131	BCIS (+) *n* = 77	*p* value	Adj. *p* value*	Adj. OR (95% CI)*
Mortality	8.7% (18)	4.6% (6)	15.6% (12)	0.006	0.008	0.24 (0.08–0.69)
Intraoperative	1.4% (3)	0% (0)	3.9% (3)	0.049	0.996	–
Postoperative	7.2% (15)	4.6% (6)	11.7% (9)	0.056	0.047	0.32 (0.11–0.99)
Hospital length of stay	16.3 ± 11.5	16.7 ± 12.4	15.7 ± 9.7	0.581		
Postoperative length of stay	14.6 ± 10.9	15.0 ± 12.0	13.9 ± 8.3	0.520		

*p-value/OR multiple regression, corrected for age

### Risk factors for BCIS

Stepwise logistic regression analysis identified the following variables as independent risk factors for the development of a BCIS: age, lack of femoral borehole and ASA status. The *R*^2^ for this model was 0.133 (Table 5).

**Table 5:** Independent risk factors for the development of a BCIS

Variables in the calculation	*p* value	OR (95% confidence interval)	*R*^2^
Age	0.003	1.05 (1.02–1.09)	0.066
Femoral bore hole	0.023	0.45 (0.22–0.90)	0.034
ASA 4	0.028	2.67 (1.11–6.40)	0.033

The following variables were also included in the analysis: Jet lavage, long stem, ASA 2

## Discussion

The BCIS is one of the most relevant intra-, but also early postoperative complications in the endoprosthetic treatment of femoral neck fractures. In our study, we were able to demonstrate that in-hospital mortality is significantly increased when a BCIS occurs. Furthermore, an increased complication rate was shown, especially for intraoperative pulmonary embolisms. Similar results were obtained in a study by Hagio et al., in which transesophageal echocardiography (TEE) was performed intraoperatively in 88 patients who received a total hip joint replacement. While embolic material was detected in 61.5% of patients with cemented hip replacement, this was only the case in 5.9% of patients with cementless hip replacement (*p* < 0.05) [7].

Olsen et al. described an incidence of BCIS of 28% [19]. Overall, however, the literature regarding the incidence of BCIS is sparse, mainly due to the fact that a precise definition of BCIS is still not available. Especially mild reactions are probably often not recognized as such and are therefore not included in studies. The incidence in our analysis was 37%; however, several factors need to be considered: supposedly, a more detailed documentation was done when a BCIS actually occurred. Furthermore, our results are based on intraoperative blood pressure values and oxygen saturation. Transesophageal echocardiography was only performed in the context of intraoperative resuscitation (*n* = 5). In this case, direct detection of thrombogenic material was possible in all patients. Furthermore, the classification of BCIS was based on Donaldson et al. This means that all blood pressure drops below 20% were not classified as BCIS and were therefore not considered as BCIS in our evaluation.

The recommendations for the handling and prophylaxis of BCIS published by the Drug Commission of the German Medical Association in 2008 include both surgical and anesthesiologic measures. From a surgical point of view, among other things, the careful treatment of the medullary cavity (e.g. by means of jet lavage), the routine insertion of a intramedullary plug, the use of a medullary drain or, alternatively, the creation of a femoral borehole and the insertion of the prosthesis stem under gentle pressure are recommended. Other guidelines focus on the cementation technique and recommend to use a distal suction catheter on top of an intramedullary plug, to insert the cement from a gun in retrograde fashion on top of the plug and to pull the catheter out as soon as it is blocked with cement [20] From the anesthesiological point of view, an intraoperative administration of 500 ml Ringer’s lactate solution is recommended in the first third of the surgical phase [21].

With regard to the surgical measures, jet lavage of the medullary cavity was performed in about 60% of the patients in our study prior to application of the cement. This mechanical cleaning process carefully prepares the medullary canal and cleans it of fat and marrow. The distal placement of a femoral borehole has been consistently applied in our clinic since 2016 and was present in about 29% of the cases examined. As a surgical measure, this borehole proved to be a statistically significant protection against the development of BCIS. Wenda et al. could already show in animal experiments in 1993 that increasing intramedullary pressure leads to the detection of increased thrombogenic material in the Vena cava [22]. In the same study, the authors were able to demonstrate in 60 patients that a femoral borehole in combination with the use of an intramedullary plug significantly weakens the circulatory reaction to an intramedullar pressure increase. An intramedullary plug, which was used in only 15% of the cases in our study, intends to prevent uncontrolled spreading of the cement in the distal part of the femur, where most of the venous drainage system of the bone marrow is localized. The relatively rare utilization of an intramedullary plug in our clinic can be explained, among other things, by the assumption that a blockage of the medullary canal by a plug can cause an additional increase in pressure when inserting the cement or the stem. While the placement of a plastic suction catheter may limit pressure peaks during application of the cement, the insertion of the stem may still significantly increase the pressure in the medullary canal, in particular when an intramedullary plug is in place. However, in the present study, the combination of an intramedullary plug and a distal borehole was present in only 1 patient. Furthermore, patients with plug did not have an increased risk of BCIS (with vs. without intramedullary plug, 34.4% vs. 37.5%, *p* = 0.736). Therefore, the effect of an intramedullary plugin combination with a distal bone hole on the occurrence of a BCIS should be further investigated. It should also be noted, that guidelines on cementing are primarily designed to maximize the survival time of cemented stems. However, from a pathophysiological point of view, the probability of sustaining a BCIS might be elevated by strictly following these recommendations. Further studies might therefore be needed to investigate potential modifications of cementing techniques that promote optimal implant survival times on the one hand, but also consider the risk of a BCIS on the other hand. Omitting the intramedullary plug (as described in the present study) and thereby exploiting the full effect of the distal femoral borehole on intramedullary pressure reduction during stem insertion might be such a modified technique. Furthermore, patient subgroups that would optimally profit from such modified techniques (limited implant survival time but a reduced risk of BCIS) should be defined in future studies.

With regard to the implanted stem (standard vs. long prosthesis stem) and its influence on the occurrence of a BCIS, no statistically significant difference could be determined based on our data. In literature, however, a higher incidence of BCIS is described when using a long prosthesis stem. In a study by Herrenbrucks et al. 34 out of 55 patients (62%) experienced cardiopulmonary complications up to coma (*n* = 2) and death (*n* = 1) after the implantation of a long stem. However, in contrast to our study the indication for the implantation of a long stem was mostly due to the presence of osseous metastases [23].

As anesthesiological measures to attenuate a possible BCIS, the optimization of the volume status and ventilation with 100% oxygen at the time of cementation is recommended. In addition, the entire surgical team is advised to pay special attention to the patient, especially in those patients with pre-existing cardiac conditions [24]. These recommendations were consistently implemented in our study during the study period. This fact explains why a clinically relevant decrease in oxygen saturation could only be observed in BCIS Grade III. Regarding blood pressure values, as defined by Donaldson et al., a gradual decrease of systolic and diastolic values with increasing BCIS severity could be observed.

The choice of surgical treatment for displaced femoral neck fractures (cemented vs. uncemented hemiarthroplasty) remains still controversial. Previous studies have already shown that the risk of death within the first postoperative day after implantation of a cemented prosthesis in femoral neck fractures is significantly increased, independent of age, gender, cognitive impairment and ASA status [25]. In a study by Parvici et al., in which almost 30,000 patients with endoprosthetic hip replacement were included, intraoperative deaths were found to occur exclusively with implantation of the cemented prosthesis. All deceased patients had a pre-existing cardiovascular disease. In the autopsies performed, pulmonary microembolisms with bone marrow were found in most cases, in some cases even methylmethacrylate particles could be found in the lungs [26]. Studies investigating the one-year mortality rate in the group of patients over 75 years of age, however, could no longer detect any significant difference between a cemented and a cementless procedure (*p* = 0.233) [27]. Olsen et al. found a total 1-year mortality rate of 29% for cemented hip endoprosthesis in all age groups. Divided into the different BCIS stages, it was higher with increasing BCIS degree [8]. In the recent study by Kristensen et al., analyzing data from the Norwegian hip fracture register, a higher mortality rate was observed at day 0 and 1 for patients with cemented compared to an uncemented hemiarthroplasty. However, and in line with previous studies, the use of cemented hemiarthroplasty decreased the risk of reoperations due to e.g. infections, periprosthetic fractures, dislocations and aseptic loosening, which all have to be seen as a devastating complication for an elderly and frail patient with a hip fracture. Furthermore, no differences in the one-year mortality rate, pain and quality of life were found in this nationwide observational study [28]. In our study, investigating cemented hemiarthroplasty only, the in-hospital mortality rate was 8.7% with a significantly higher mortality in patients with BCIS. Intraoperative deaths occurred only in the BCIS (+) group.

Several statistically significant risk factors for the occurrence of BCIS could be identified from our data. As variables which cannot be influenced by anesthesia or surgery, increasing age and ASA status were shown to be risk factors for BCIS. The creation of a femoral borehole as an easy to perform surgical measure before application of the cement proved to be a protective factor with regard to the development of a BCIS. The *R*^2^ of this risk factor analysis was comparatively low at 0.133. Accordingly, future studies should concentrate on the identification of further factors favoring BCIS. In the literature, other risk factors include the presence of COPD and the preoperative use of diuretics or warfarin as long-term medication [8]. Studies have also shown that a volume deficit prior to cement application can have adverse effects, particularly if the ventricular filling mechanism is restricted, e.g. by atrial fibrillation, or right ventricular function is impaired [24].

The present study has several limitations. Due to the retrospective study design not all data could be collected completely. The number of patients included was limited in particular by the fact that not all anesthesia protocols clearly indicated the time of cement application. Thus, 181 patients could not be included in the study. However, a comparative analysis of the included and excluded patients did not reveal any statistically significant differences in the demographic and outcome parameters of the two groups, which makes a relevant bias unlikely. Furthermore, the evaluation was carried out over a period of 9.5 years, during which the patients were treated by different anesthetists and trauma surgeons. However, this reflects the clinical routine very well. In future studies addressing BCIS, a prospective assessment of BCIS and parameters relevant to BCIS should be carried out. For example, the influence of a PEEP increase before cement application on the occurrence of BCIS would be an interesting aspect of further studies.

## Conclusion

The occurrence of BCIS is frequently observed, in some cases serious event. Therapy can ultimately only be carried out symptomatically with a stabilization of the patient's vital functions so that it is all the more important to consider the possibilities of preventive measures. On the basis of our retrospectively collected data, we were able to prove that the in-hospital mortality is significantly increased when BCIS occurs. ASA status and age were identified as risk factors for a cement reaction. The creation of a distal femoral borehole for pressure reduction in the medullary cavity was shown to be a significant protective factor against BCIS.
